# Epidemiological characterization of viral etiological agents of the central nervous system infections among hospitalized patients in Egypt between 2016 and 2019

**DOI:** 10.1186/s12985-023-02079-y

**Published:** 2023-08-02

**Authors:** Wael H. Roshdy, Ahmed Kandeil, Manal Fahim, Nourhan Y. Naguib, Gehad Mohsen, Shaymaa Shawky, Marwa M. Abd El-Fattah, Amel Naguib, Azza Salamony, Yara I. Shamikh, Mahmoud Moawad, Nancy El Guindy, Mohamed K. Khalifa, Eman Abbas, Ramy Galal, Mohamed Hassany, Mohamed Ibrahem, Rabeh El-Shesheny, Noha Asem, Amr Kandeel

**Affiliations:** 1 Department of Virology, Central Public Health Laboratories, Ministry of Health, Cairo, 11613 Egypt; 2 Center of Scientific Excellence for Influenza Viruses, National Research Centre, Giza, 12622 Egypt; 3 Department of Epidemiology and Surveillance, Ministry of Health, Cairo, 11613 Egypt; 4 Virology Department, Egypt Centre for Research and Regenerative Medicine, ECRRM, Cairo, 11517 Egypt; 5 Department of Pathology, National Cancer Institute, Cairo University, Cairo, Egypt; 6 Public Health Initiative, Ministry of Health and Population, Cairo, Egypt; 7 National Hepatology and Tropical Medicine Research Institute, Ministry of Health and Population, Cairo, Egypt; 8 Department of Medical Biochemistry and Molecular Biology, Faculty of Medicine, Cairo University, Cairo, 12613 Egypt; 9 Department of Public Health, Faculty of Medicine, Cairo University, Cairo, 12613 Egypt; 10 Ministry of Health and Population, Cairo, Egypt

## Abstract

Viral infections of the central nervous system (CNS) are common worldwide and result in considerable morbidity and mortality associated with neurologic illness. Until now, there have been no epidemiologic data regarding viruses causing aseptic meningitis, encephalitis, and CNS infections in Egypt. We investigated 1735 archived cerebrospinal fluid samples collected from Egyptian patients between 2016 and 2019 and performed molecular characterization for infection for12 different viruses: herpes simplex viruses 1 and 2 (HSV-1 and HSV-2), varicella-zoster virus (VZV), Epstein–Barr virus (EBV), cytomegalovirus (CMV), human herpesviruses 6 and 7 (HHV-6 and HHV-7), human enteroviruses (HEVs), human parechovirus (HPeV), parvovirus B19 (B19V), adenovirus (AdV), and mumps virus (MuV). All included samples were negative for bacterial infection. Our results indicated a relatively high prevalence of viral infection, with HEVs being the most prevalent viruses, followed by HSV-1, EBV, and then HSV-2. The highest prevalence was among male patients, peaking during the summer. Data obtained from this study will contribute to improving the clinical management of viral infections of the CNS in Egypt.

## Introduction

Infection of the central nervous system (CNS) by several viruses can cause neurologic illnesses, including meningitis, encephalitis, encephalomyelitis, and others. The estimated number of viral CNS infections annually is approximately 20–30 per 100,000 individuals [1]. Viral meningitis is the most common of these CNS infections, particularly in young children, in whom it is caused by enteroviruses, herpes simplex viruses, mumps, and measles. The prevalence of viral CNS infections varies with geographic area, sex, and age. CNS infections may differ in their severity, signs, symptoms, and etiology [2] and can occur across a wide age range, although they are more common in children. Physicians must be sufficiently familiar with these different outcomes and causes of viral infection in order to give a proper prognosis and to prescribe the appropriate treatment with the proper timing, thereby avoiding unnecessary antibiotic usage and preventing negative effects on the CNS.

The etiology of CNS infections may involve pathogens, such as bacteria, fungi, protozoa, and viruses, or non-infectious causes, such as physical injury, tumors, or the use of certain drugs [3]. However, viral agents are reported to cause more infections annually when compared to other pathogens. The incidence of viral meningitis has been reported to be approximately 28 cases per 100,000 in children younger than 14 years [4] and approximately 8 cases per 100,000 in adults [5]. Also, an incidence of 7.6 cases of aseptic meningitis per 100,000 persons was recorded and most of cases caused by viruses [1, 5].

Virus families associated with CNS infections include the *Paramyxoviridae* (e.g., measles virus and mumps virus [MuV]) [6], the *Togaviridae* (e.g., eastern, western, and Venezuelan equine encephalitis viruses) [7, 8], the *Adenoviridae* (adenovirus [AdV]) [9], the *Flaviviridae* (e.g., West Nile virus) [10], the *Parvoviridae* (e.g., parvovirus B19 [B19V]) [11], the *Herpesviridae* (e.g., herpes simplex viruses types 1 and 2 [HSV-1 and HSV-2], varicella-zoster virus [VZV], Epstein–Barr virus [EBV], cytomegalovirus [CMV], and human herpesviruses 6 and 7 [HHV-6 and HHV-7]) [12], 13, and the *Picornaviridae* (e.g., human parechovirus [HPeV] and human enteroviruses [HEVs]) [14].

Viral infections of the CNS are acquired through ingestion, breathing, or insect bites. After entering the body, the viruses infect the vascular endothelial cells, directly cross the blood–brain barrier, or enter through the unprotected parts of the blood–brain barrier [15]. At this stage, inflammation can occur in different areas of the CNS [15].

Viral infection of the CNS can be diagnosed by molecular detection of viral nucleic acid in the cerebrospinal fluid (CSF). A positive PCR test for a virus soon after the onset of disease can save a patient with mild symptoms from an unnecessary hospital admission [6]. However, the limitations of the molecular diagnostic units in low-to-middle-income countries can impair the proper diagnosis of CNS infections.

As a consequence of the misuse and abuse of antimicrobial agents, several multidrug-resistant forms of bacterial meningitis with high prevalence rates have been recorded [16]. To our knowledge, however, few studies have characterized the epidemiologic distribution of viral infections of the CSF in Egypt [16–18]. Therefore, the objectives of this study were to determine the prevalence of viral CNS infection among patients admitted to hospital in Egypt between 2016 and 2019, to identify the causative agents, and to investigate the distribution with respect to sex, age, and governorate.

## Materials and methods

The study included a total of 1735 archived CSF samples collected from patients who tested negative for bacteriologic CNS infection and who were admitted to fever hospitals in different Egyptian governorates between February 2016 and February 2019 based on a case definition of meningitis and encephalitis. All patients experienced a sudden onset of fever with a temperature higher than 38 °C, together with one or more of the following: neck stiffness, confusion, headache, vomiting, drowsiness, seizures, difficulty in speaking, sore throat, and other signs of meningeal irritation and rash [12]. As part of the routine clinical care, a lumbar puncture was performed, and CSF samples (4–5 mL each) were drawn before the initiation of any antibiotic treatment prescribed by the hospital clinicians. The samples were preserved in liquid nitrogen at the hospitals then transported to the Central Public Health laboratories (CPHL) in Cairo, Egypt.

In this study, archived CSF samples were considered for viral identification by PCR if they tested negative for bacteriologic meningitis and encephalitis, the protein levels detected by clinical chemistry testing were lower than 100 mg/dL, and the lymphocyte counts were between 5 and 1000 cells/µL.

Multiplex quantitative reverse transcriptase PCR (RT-qPCR) was used to screen for RNA and DNA viruses in nucleic acid extracted from the CSF samples. The 12 targeted viruses were HSV-1, HSV-2, VZV, EBV, CMV, HHV-6, HHV-7, HEVs, HPeV, B19V, AdV, and MuV.

A volume of 400 μL of each CSF sample was used for total nucleic acid extraction with a Qiagen EZ1 Virus Mini Kit v2.0. After the nucleic acid had been purified, it was recovered in 60 μL of elution buffer. The FTD Neuro 9 assay (Fast Track Diagnostics), a four-tube multiplex PCR system, was used to detect the 12 viruses most responsible for causing meningitis/encephalitis, along with an internal control. The multiplex PCR technique was based on the TaqMan system [19]. For each sample, 10 μL of the extracted nucleic acid was processed on an Applied Biosystems 7500 RT-PCR System (Thermo Fisher Scientific, Waltham, MA) and the results were collected and analysed.

### Statistical analysis

**Fig. 1:**
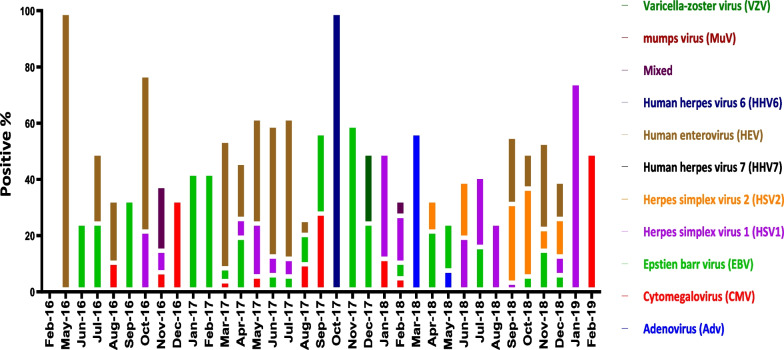
Monthly viral detection among CNS infected hospitalized patients with CNS infections during the period from February 2016 to February 2019 in Egypt. Arranged bar chart showing the percentage of positive cases of each specific virus monthly

Chi-square was used to compare seropositivity rates within categorical variables. *P* value < 0.05 was considered statistically significant. Logistic regression was used to calculate adjusted odds ratios using all variables that were significant in bivariate analysis (Fig. 1).

## Results

Of the 1735 patients for whom CSF samples were screened, 330 (19.0%) tested positive for viral infection by molecular methods. Encephalitis was diagnosed in 247 (74.8%) of these virus-positive patients, and meningitis was diagnosed in the remaining 83 (25.2%) (Table 1). Detection rates differed significantly by year, place of residence, and sex (Table 1). Viral infections of the CNS were detected regardless of the season. Highly significant differences were observed in the detection rates for viral infection by year (*P* < 0.0001) (Table 1).

**Table 1:** Demographic and health data of the study participants

Variable	No. of samples collected (%) (*N* = 1735)	No. of virus- positive samples (%) (*N* = 330)	*P*
Year			
2016	261 (15.0)	46 (13.9)	0.0001
2017	657 (37.9)	134 (40.6)	
2018	701 (40.4)	144 (43.6)	
2019	116 (6.7)	6 (1.8)	
Age (years)			
< 1–10	781 (45.0)	145 (43.9)	0.1676
11–20	247 (14.2)	49 (14.8)	
21–30	192 (11.1)	41 (12.4)	
31–40	161 (9.3)	32 (9.7)	
41–50	74 (4.3)	12 (3.6)	
51–60	117 (6.7)	26 (7.9)	
61–70	87 (5.0)	17 (5.2)	
> 70	76 (4.4)	8 (2.4)	
Sex			
Female	668 (38.5)	111 (33.6)	0.0451
Male	1067 (61.5)	219 (66.4)	
Place of residence			
Greater Cairo Region	857 (49.4)	177 (53.6)	0.0009
Alexandria Region	320 (18.4)	68 (20.6)	
Delta Region	227 (13.1)	32 (9.7)	
Upper Egypt Region	295 (17.0)	40 (12.1)	
Suez Canal Region	36 (2.1)	13 (3.9)	
Disease			
Encephalitis	1237 (71.3)	247 (74.8)	0.1130
Meningitis	498 (28.7)	83 (25.2)	
Outcome			
Death	228 (13.1)	43 (13.0)	0.80
Discharged	1450 (83.6)	275 (83.3)	
Unknown	57 (3.3)	12 (3.6)	

Patients living in Greater Cairo were most vulnerable to viral infection, accounting for 53.6% of the 330 virus-positive cases (n = 177). They were followed by patients in the Alexandria region, who accounted for 20.6% of the positive cases (n = 68), and those in Upper Egypt, who accounted for 12.1% (n = 40). Of the 330 virus-positive patients, 219 (66.4%) were male. The median age of the virus-infected patients was 10 years. The fatality rate was 13% for the patients with viral meningitis (Table 1).

Fever was the most commonly reported symptom among patients with viral infection of the CNS, being reported in 95.7% of cases. This was followed by altered consciousness (in 64.3% of cases), vomiting (in 63.1%), seizures (in 59.9%), headache (in 50.8%), neck rigidity (in 36.1%), irritability (in 32.0%), and a bulging fontanel (in 12.4%) (Table 2). The etiologic profile of the viral infections showed that 9% of the patients (n = 30) had a mixed infection, whereas the remainder were infected with only a single virus (Table 2).

**Table 2:** Symptoms of study participants with viral infections of the central nervous system

		Viral PCR binary				*P*	Odds Ratio	95% Confidence Interval	
		Negative		Positive				Lower	Upper
		n	%	n	%				
Fever	Yes	1331	96.9	311	95.7	0.289	1.393	0.753	2.579
	No	43	3.1	14	4.3				
Neck rigidity	Yes	374	36.0	86	36.1	0.968	0.994	0.741	1.333
	No	665	64.0	152	63.9				
Vomiting	Yes	737	61.8	173	63.1	0.675	0.944	0.719	1.238
	No	456	38.2	101	36.9				
Headache	Yes	472	47.3	121	50.8	0.325	0.868	0.654	1.151
	No	526	52.7	117	49.2				
Seizures	Yes	685	58.1	160	59.9	0.575	0.925	0.706	1.213
	No	495	41.9	107	40.1				
Altered consciousness	Yes	677	62.9	157	64.3	0.677	0.940	0.704	1.256
	No	399	37.1	87	35.7				
Bulging fontanel	Yes	74	8.6	25	12.4	0.094	0.664	0.410	1.075
	No	785	91.4	176	87.6				
Irritability	Yes	314	32.3	70	32.0	0.922	1.016	0.742	1.391
	No	658	67.7	149	68.0				

**Fig. 2:**
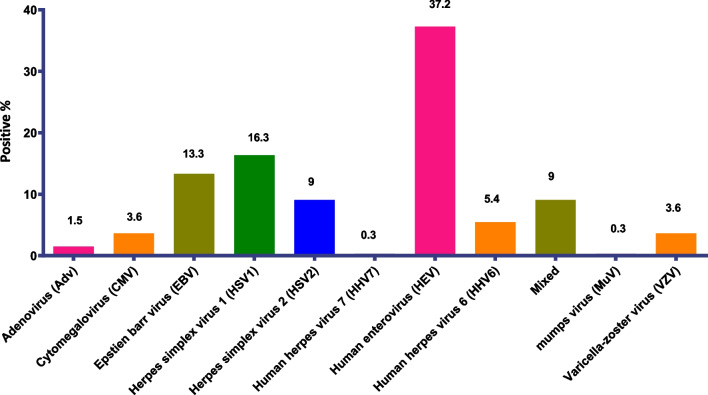
Distribution of different virus types among virus-positive cases of CNS infected hospitalized patients in Egypt between 2016 and 2019

HEV was the predominant virus, being present in 37.3% of the virus-positive samples (n = 123), with a particularly high circulation rate during the summer. This was followed by HSV-1 in 16.4% of the samples (n = 54), EBV in 13.3% (n = 44), HSV-2 in 9.1% (n = 30), and HHV-6 in 5.5% (n = 18). CMV, HHV-7, MuV, and B19V occurred singly and in mixed infections with HEV, EBV, or VZV. HPeV was not found in any sample. The incidence rates for the different viruses in the study cases are shown in Fig. 2.

Among the viral encephalitis cases, infections by HSV-1, HSV-2, VZV, EBV, CMV, HHV-6, HEV, and AdV were detected. Among the viral meningitis cases, except for HPeV all of the characterized viral agents were detected.

## Discussion

Viral infection of the CNS is one of the most important causes of neurologic illness. The circulatory patterns of viruses that cause CNS infections, i.e., meningitis, encephalitis, and others, are country dependent, and there is only limited epidemiologic data on the prevalence of viral CNS infections in Egypt. We used multiplex RT-qPCR to screen CSF samples for 12 viral pathogens, after confirming that the samples were negative for bacterial CNS infection. The multiplex PCR methodology represents a milestone in the rapid detection of RNA and DNA viruses in CSF, easing the diagnosis of infection and the prescription of antivirals both locally and worldwide.

Of the 1735 patients who had neurologic symptoms, 330 were confirmed to be positive for a viral pathogen. The mortality associated with viral infection of the CNS is higher than that associated with viral infections of other systems [20]. Similar to previous global reports, the case fatality rate among the virus-positive cases in our study group was 13%.

In consistent with previous studies{Kohil, 2021 #380}, viral infections were most common in young children (< 1–10 years) with 43.9% but is seen across all age groups and this could be due to a combination of immunological factors, as well as, close contact with other children, and poor hygiene habits.

The predominance of male patients with viral CNS infection in our study of cases in Egypt was consistent with the findings of other studies in different countries [20–22]. HEV was the most predominant virus in our study, being present in 37.3% of virus-positive samples, which is similar to the results reported by others [23, 24]. In our study, the frequency of infection with HEV was highest in the summer months and in patients between 4 and 17 years of age, which is also consistent with other reports [25].

Akkaya et al. [24] also found HEV to be the leading causative agent of CNS infection and to have the highest prevalence in children and in summer, although they reported AdV to be the second most predominant virus, whereas in our study AdV was detected in only five of the 330 virus-positive cases (1.5%). In our study, HSV-1, EBV, and HSV-2 followed HEV in prevalence overall, although HSV-2 and VZV were reported to be prevalent in some regions.

Kupila et al. [5] also reported HEV to be the leading cause of viral meningitis, followed by HSV-2, whereas we found HSV-1 to be the second most predominant virus, and also found HSV-1 to be one of the most common viral causes of meningitis.

VZV was reported by Kupila et al. to be the third leading cause of viral CNS infection [5], but in our study it ranked seventh, being found in only 12 of the 330 virus-positive cases (3.6%), with EBV being the third most prevalent virus. Our findings are consistent with those of [26], who reported EBV to be one of the main causes of single-virus and mixed-virus encephalitis and aseptic meningitis,

In our study, HPeV was not detected in any sample, which is in marked contrast to previous studies [27–29] that found HPeV to be the second most predominant virus causing CNS infection and an important cause of sepsis-like illness in infants. Our results may be partly explained by the finding of Harvala et al. [30] that HPeV is less frequent in sepsis or meningitis in neonates than in older patients but is associated with gastroenteritis.

Although MuV was the most common cause of viral meningitis a few decades ago, we detected it in only one sample in our study. This may be a consequence of the introduction of measles–mumps–rubella vaccine in Egypt in the 1990s.

The etiologic diagnosis was achieved in 43% of the patients with meningitis and in 17% of those with encephalitis by RT-qPCR, which is an important method for the rapid detection of RNA and DNA viruses in CSF. RT-PCR has demonstrated a specificity range of 94%–100%, and these multiplex RT-PCR assays can supplement other diagnostic tests.

In our study, 30 patients had mixed infections involving CMV, HHV-7, MuV, B19V, and/or HEV, EBV, or VZV. Dyachenko et al. [31] also reported mixed infections involving more than one virus.

This study has some limitations, including the exclusion of the West Nile virus, Japanese encephalitis virus, Tick-borne encephalitis virus, and Zika virus from our screening due to a lack of diagnostic tools. Data obtained from this study will contribute to improving the clinical management of viral infections of the CNS in Egypt.

## Data Availability

All data generated or analyzed during this study are included in this published article.
